# Radiation-Induced Nasopharyngeal Fibrosis Resulting in a Difficult Airway: A Case Report

**DOI:** 10.7759/cureus.79130

**Published:** 2025-02-16

**Authors:** Mai Akazawa, Morihiro Shimizu, Yoshihisa Fujino, Hiromi Kato

**Affiliations:** 1 Department of Comprehensive Surgery, Shiga University of Medical Science, Shiga, JPN; 2 Department of Anaesthesiology, Shiga University of Medical Science, Shiga, JPN

**Keywords:** cancer of the head and neck, difficult airway management, general anesthesia, mask ventilation, nasopharyngeal cancer, radiation-induced fibrosis, radiation therapy, tracheal intubation

## Abstract

Patients with head and neck cancer often experience airway complications. This case highlights a patient with nasopharyngeal cancer post-radiotherapy (RT), who presented with a difficult airway (DA). An 83-year-old woman, scheduled for a partial left lower lobe pneumonectomy, had received RT for nasopharyngeal cancer one year prior. Physical examination revealed trismus, limited neck movement, Mallampati class IV, and grade III on the upper lip bite test. Bronchoscopy showed mucous membrane irregularities in the nasopharynx, along with an edematous epiglottis and vocal cords. After general anesthesia induction, both mask ventilation and McGrath™ intubation (Covidien Inc., Tokyo, Japan) attempts failed. Lifting the patient's tongue manually allowed visualization of the vocal cords via a fiberoptic bronchoscope, enabling successful intubation. Patients who have undergone head and neck radiotherapy (HNRT) are at increased risk of DAs due to radiation-induced fibrosis (RIF) of pharyngeal soft tissues.

## Introduction

Head and neck cancer affects over 650,000 people globally each year, with head and neck radiotherapy (HNRT) being a primary treatment option [1]. As prognosis improves, more patients are requiring general anesthesia for unrelated treatments following HNRT. HNRT increases the risk of difficult airway (DA) management (DAM) [2,3]. Radiation therapy causes various changes in the airway, one of which is radiation-induced fibrosis (RIF). RIF causes anatomical changes to the airway. Oishi et al. reported a case in which radiotherapy (RT) caused supraglottic changes, making tracheal intubation difficult [4]. This case report describes a patient who developed a "cannot ventilate, cannot intubate" (CVCI) scenario during general anesthesia induction due to RIF following RT for nasopharyngeal carcinoma (NPC).

## Case presentation

**Figure 1:**
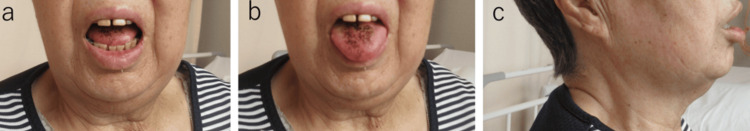
Preoperative physical examination (a) The patient had trismus with a maximum mouth opening of only 20 mm; (b) Her Mallampati classification was grade IV; (c) Her cervical mobility was restricted.

**Figure 2:**
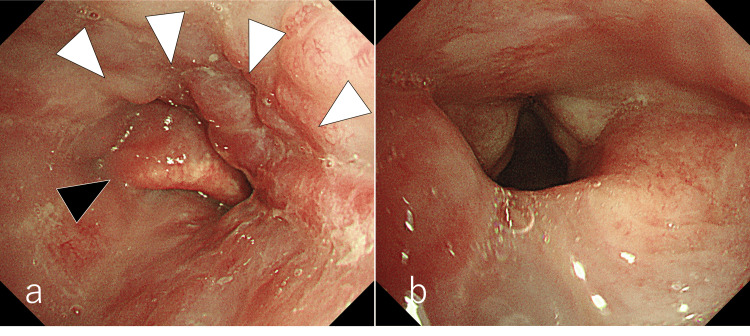
Preoperative bronchoscopy examination (a) The epiglottis (▲) was edematous with mild mucosal irregularity in the epiglottis valley, suggestive of scarring (△) from radiotherapy; (b) The vocal cords were also edematous, but the movement was unremarkable.

The patient, an 83-year-old Asian woman, was diagnosed with stage IV NPC (T3N2bN0) one year prior. The tumor occupied the nasopharynx and extended to the skull base. She received 70 Gy of RT over three months, during which she developed stomatitis, resolving post-RT but leaving persistent oral pain. The irradiated area extended from the oral cavity to the cervical esophagus, including the spinal cord. A chest computed tomography (CT) scan performed eight months after RT revealed a 6 mm nodule in the S6 region of the lower lobe of the left lung. A month later, a partial left lung resection was scheduled. Preoperative examination revealed severe trismus (maximum mouth opening of 20 mm) and restricted neck movements (Figures 1a, 1c). The patient had a Mallampati class IV airway (Figure 1b), a grade III upper lip bite test, and a thyromental distance (TMD) of <60 mm. She could not protrude her lower jaw due to intraoral pain. No signs of snoring or sleep apnea were observed. Bronchoscopy revealed an edematous epiglottis and vocal cords, with mild mucosal irregularity in the epiglottic valley, likely a scar from RT (Figure 2). Vocal cord movement was normal. No apparent stomatitis was observed. The examination, performed in the supine position under sedation with spontaneous breathing, allowed the straightforward insertion of a fiberoptic bronchoscope from the oral cavity into the trachea. Preoperative chest radiography and CT revealed no tracheal deviation or stenosis. Blood tests, pulmonary function tests, and electrocardiography were normal.

General anesthesia with an epidural was planned. After catheter placement at the thoracic 5-6 level, general anesthesia was induced. Although mouth opening and neck flexion were limited, we thought it would be possible to use an oral airway or McGrath™ MAC video laryngoscopy (Covidien Inc., Tokyo, Japan) and chose rapid induction. The patient was positioned supine, and oxygen at 5 L/min was administered. Remifentanil (0.7 mg/h) and remimazolam (4 mg, then 40 mg/h continuously) were infused. Rocuronium (40 mg) was administered, but mask ventilation proved difficult. An oral airway was attempted, but the tip struck the posterior pharyngeal wall and could not be placed in the correct position. Intubation using McGrath™ failed (Cormack grade IV), and oxygen saturation (SpO_2_) dropped to 89%, but mask ventilation with a two-hand mask hold restored it to 100%. Reattempted intubation with McGrath™ and a bronchoscope was unsuccessful due to a stiff epiglottic vallecula, preventing laryngeal exposure. Due to limited mouth opening, the Pentax-AWS Airwayscope™ (Hoya Corporation, Tokyo, Japan) could not be inserted. There were no McGrath™ X-blades or AWS thin blades in our hospital. Given the difficulty with intubation and persistent tongue root depression, it was thought that intubation was nearly impossible under the administration of muscle relaxants. Since the patient had been able to undergo fiberoptic bronchoscopy without any problems while breathing spontaneously, we considered awake intubation. However, lifting the tongue manually allowed visualization of the epiglottis and vocal cords with a bronchoscope. McGrath™ was not in use at this time. Using fiberoptic guidance, a 7.0 mm single-lumen endotracheal tube was successfully placed, and a bronchial blocker was inserted into the left bronchus for one-lung ventilation.

The patient underwent a partial left pneumonectomy as scheduled. After the procedure, the tracheal tube cuff was removed, and a leak was observed. An attempt to insert a nasopharyngeal airway for extubation failed due to a tip hit. Fiberscopic observation revealed stenosis of the mesopharynx, preventing passage of the airway. The patient was extubated after full arousal without the airway, and stable spontaneous breathing was confirmed. There was no hoarseness, and respiratory status remained stable. The patient was transferred to the high-care unit. She continued to progress without further complications and was discharged on postoperative day 8.

## Discussion

This case highlights two key points. First, patients after HNRT are more likely to require DAM due to RIF. Second, patients who have undergone NPC/RT are at a higher risk of DA, requiring careful anesthesia planning, including preservation of spontaneous breathing and fiberoptic intubation.

HNRT is a known independent predictor of difficult mask ventilation and tracheal intubation [2,3], primarily due to RIF of the upper airway [5]. HNRT-induced fibrosis can cause complications such as radiation-induced trismus, temporomandibular joint fibrosis, fibrosis of the hyoid muscle, neck dystonia, neck stiffness, shoulder joint dysfunction, skin fibrosis, and facial lymphedema [1,6]. RIF results from an abnormal wound healing response, where radiation injury triggers inflammation and fibroblast differentiation into myofibroblasts, resulting in excessive myofibroblast proliferation, collagen production, and decreased vascularity, which cause fibrosis and reduced tissue compliance [7]. Kannan and Arul Ponni reported that patients who received HNRT suffered from long-term dysphagia. In particular, they reported that patients who received 45 Gy or more had a slower recovery of swallowing function than patients who received less than 45 Gy [8]. Thus, it is expected that the severity of RIF will vary depending on the radiation dose, duration, and site. However, predicting the severity and prognosis of RIF is challenging [9], and no definitive treatments or preventive strategies exist [1]. Radiographic fibrotic changes typically occur within the first year post-treatment and worsen over time [1]. Kent et al. reported trismus developing gradually nine weeks post-RT, progressively worsening for up to four years [9]. Therefore, fibrosis can continue to develop long after RT.

RIF can complicate supraglottic apparatus (SGA) insertion [10,11], primarily due to pharyngeal narrowing and restricted neck retroflexion. These anatomical changes make proper SGA placement and effective ventilation difficult. Giraud et al. found that, in patients who underwent cervical and oral RT [10], those with cervical RT had more difficulty with SGA placement and ventilation, suggesting a greater anatomical impact on the upper airway. This difficulty with SGA raises concerns for post-HNRT patients, as these airways, crucial for rescue in DAM, may be less effective.

Patients who have undergone HNRT, particularly those with NPC, are at higher risk for DA. A study on 134 post-RT patients found a higher proportion of Mallampati class III/IV (77% vs. 67%, p = 0.07) and restricted neck motion (35% vs. 11%, p < 0.001) than in pre-RT patients [5]. However, no significant differences were found in mask ventilation difficulty (2% vs. 2%, p = 1.00), difficult tracheal intubation (DTI) (2% vs. 5%, p = 0.34), or visual field during laryngeal deployment. In comparison, general studies report difficulty in mask ventilation (5%) [11] and laryngeal deployment by laryngoscopy (5.8%) [12]. This suggests that DA rates in post-HNRT patients may not differ significantly from those in general surgery. A retrospective study involving 150 post-NPC/RT patients reported a 28% incidence of DTI [13], which is notably higher than in previous studies. DTI occurred despite bronchoscopy and video laryngoscopy. The probability of DTI was 7.1 times higher in patients with concurrent cervical mobility restriction and trismus. Additionally, non-White patients were more likely to experience DTI. A major factor contributing to this high DA rate is that standard NPC/RT affects most of the upper airway [6]. Therefore, general anesthesia in post-NPC and cervical RT patients requires careful planning, including preserving spontaneous breathing and fiberoptic intubation.

According to Kheterpal et al.'s risk index [2], the probability of both mask ventilation difficulty and DTI in this case was 3.31% (class V, seven risk factors). Given this, routine awake intubation for all post-NPC patients who have undergone RT remains controversial. In this case, the patient exhibited trismus, restricted cervical mobility, and residual oral pain from mandibular protrusion. Bronchoscopy revealed radiation-induced scarring in the glottic valley, suggesting upper airway inflammation from RT. These findings indicated a risk of airway obstruction from RIF. After administering rocuronium, mask ventilation failed. An oral airway and video laryngoscope could be inserted, but, contrary to the authors' expectations, pharyngeal fibrosis prevented them from being properly positioned, and the tongue could not be released. Fortunately, in this case, two-person mask ventilation was successful, and restoration of spontaneous breathing or keeping invasive airways was not required. If both mask ventilation and tracheal intubation are not possible, immediate restoration of spontaneous breathing or surgical airway management, including surgical cricothyroidotomy, large-bore cannula cricothyroidotomy, or surgical tracheostomy, is required according to the DAM algorithm [14]. However, not all RIF patients require invasive airways; accurately assessing DA risk in RIF patients is a challenge for anesthesiologists. A study by Huang et al. involving 150 post-NPC/RT patients found that DTI could be predicted preoperatively by anesthesiologists, but sensitivity and specificity were low (54.8% and 63.9%, respectively) [13]. No significant differences in preoperative physical assessments (Mallampati score, TMD, limited neck extension, or mouth opening) were observed between DTI and non-DTI groups. Notably, patients who died (1/150) or required extracorporeal membrane oxygenation (1/150) were not predicted to be at high risk for DTI preoperatively. This highlights that physical and imaging findings may not reliably predict airway management challenges in post-NPC/RT patients. In this case, tracheal intubation was successful by manually lifting the tongue. This is a well-known method to facilitate oral intubation [15]. Although further studies are needed to determine whether it is effective, this method may be considered as one of the options for DAM in patients with RIF. Future research should focus on improving risk assessment, considering factors like mandibular motion, mucosal changes, and the RT irradiation site.

## Conclusions

We encountered a case of a post-NPC/RT patient who developed CVCI after receiving muscle relaxants during general anesthesia induction. HNRT often leads to DA due to RIF, which can severely compromise the airway, particularly in post-NPC/RT patients. However, the severity of RIF varies from person to person, and it is impossible to predict which parts of the airway are affected. It is difficult to accurately assess the difficulty of securing the airway with RIF based solely on perioperative physical findings and subjective symptoms. Therefore, careful anesthesia planning is essential in such cases, with special consideration given to awake intubation and fiberoptic intubation.
